# Microbial Community Shifts in Response to Acid Mine Drainage Pollution Within a Natural Wetland Ecosystem

**DOI:** 10.3389/fmicb.2018.01445

**Published:** 2018-06-27

**Authors:** Oscar E. Aguinaga, Anna McMahon, Keith N. White, Andrew P. Dean, Jon K. Pittman

**Affiliations:** ^1^School of Earth and Environmental Sciences, Faculty of Science and Engineering, University of Manchester, Manchester, United Kingdom; ^2^School of Science and the Environment, Faculty of Science and Engineering, Manchester Metropolitan University, Manchester, United Kingdom

**Keywords:** acid mine drainage, bacterial community, metabolic prediction, metal pollution, microbial ecology, wetlands, 16S rRNA gene amplicon sequencing

## Abstract

Natural wetlands are known to play an important role in pollutant remediation, such as remediating acid mine drainage (AMD) from abandoned mine sites. However, many aspects of the microbiological mechanisms underlying AMD remediation within wetlands are poorly understood, including the role and composition of associated microbial communities. We have utilized an AMD-polluted river-wetland system to perform rRNA sequence analysis of microbial communities that play a role in biogeochemical activities that are linked to water quality improvement. Next-generation sequencing of bacterial 16S rRNA gene amplicons from river and wetland sediment samples identified variation in bacterial community structure and diversity on the basis of dissolved and particulate metal concentrations, sediment metal concentrations and other water chemistry parameters (pH and conductivity), and wetland plant presence. Metabolic reconstruction analysis allowed prediction of relative abundance of microbial metabolic pathways and revealed differences between samples that cluster on the basis of the severity of AMD pollution. Global metabolic activity was predicted to be significantly higher in unpolluted and wetland sediments in contrast to polluted river sediments, indicating a metabolic stress response to AMD pollution. This is one of the first studies to explore microbial community structure dynamics within a natural wetland exposed to AMD and our findings indicate that wetland ecosystems play critical roles in maintaining diversity and metabolic structure of sediment microbial communities subject to high levels of acidity and metal pollution. Moreover, these microbial communities are predicted to be important for the remediation action of the wetland.

## Introduction

Pollution from abandoned mine sites is a major environmental problem that has deleterious consequences for aquatic and terrestrial ecosystems (Bridge, 2004). Freshwater resources are negatively impacted by mining contamination (Johnson and Hallberg, 2005), reducing the value of water for agriculture, recreation, or industry, and rendering it unsafe for human consumption (Tripole et al., 2006). Release of metals from exposed rock and tailings plus uncontrolled drainage of metal-contaminated water is a common challenge from disused mines. For many mines, including coal mines, the drainage is also highly acidic. This acid mine drainage (AMD) is generated when metal sulfide minerals, mainly pyrite, are oxidized in the presence of water and accelerated by the action of chemolithotrophic bacteria. This generates acidity and mobilizes large concentrations of iron (Fe), and other toxic trace metals and metalloids, depending on the composition of the exposed rock (Robb, 1994; Mason, 2002). AMD pollution from abandoned mines worldwide therefore is a major problem that needs to be mitigated and managed (Hedin et al., 1994; Mayes et al., 2009b).

The characterization of microorganisms in such polluted environments is important in order to understand the impacts of AMD on microbial ecology and evolution, to identify microorganisms that may have bioremediation properties, and to understand the mechanisms of microbial AMD tolerance and remediation (Méndez-García et al., 2015; Chen et al., 2016; Huang et al., 2016). The ability to identify bacterial taxa and quantify bacterial abundance and diversity in AMD environments has been significantly advanced through the use of next-generation sequencing and metagenomics tools (Amaral-Zettler et al., 2011; Kuang et al., 2013; Brantner and Senko, 2014; Liu et al., 2014). In particular, this has allowed bacterial community structure and water quality status to be correlated with functional characteristics (Chen et al., 2015; Hua et al., 2015; Kuang et al., 2016). These analyses revealed that while AMD environments have significantly reduced species richness and diversity, they exhibit a high abundance of acidophilic taxa. Moreover, bacterial diversity between different AMD environments can be predicted in part by physicochemical characteristics, especially pH (Kuang et al., 2013; Liu et al., 2014). These studies also demonstrate that AMD tolerant bacteria use multiple metabolic processes to survive these environments, such as high expression of genes encoding iron and sulfur oxidation enzymes, multiple modes of carbon and nitrogen metabolism, and high abundance of metal transporter genes to withstand metal stress. Furthermore, there is evidence that transcriptional profiles of bacterial communities alter in response to different AMD water quality characteristics (Chen et al., 2015; Kuang et al., 2016).

Microbial community structures have been examined in mine tailings, AMD water and sediment, and biofilms from mine sites (Huang et al., 2016). However, very few previous AMD studies have used large-scale sequencing approaches to examine bacteria associated with wetland plant rhizosphere sediments (Diaby et al., 2015), and to our knowledge, none have characterized AMD-exposed natural wetlands in direct comparison to river sediment without wetland plants. Wetlands can show substantial resilience to highly metal-rich and acidic waters over very long periods of time; for example, natural wetlands that have been studied in Ireland, United Kingdom, and United States have been found to tolerate very high acidity (sometimes pH < 3), high dissolved metal concentrations (such as >200 mg L^-1^ of Fe) over many decades, indicating long-term adaptation to AMD stress (Beining and Otte, 1996; August et al., 2002; Dean et al., 2013). Indeed natural and constructed wetlands are often considered as a passive, low-maintenance approach to mine drainage remediation by slowing and reducing drainage run-off, enhancing bulk uptake of metals into biomass, and by providing organic carbon to maintain rhizosphere bacteria to drive redox reactions including sulfate reduction (Sheoran and Sheoran, 2006; Mayes et al., 2009a; Dean et al., 2013). These reactions will in turn reduce concentrations of dissolved metals through the formation of metal precipitates and sedimentation, and promote alkalization (Hallberg and Johnson, 2005). However, it is argued that wetlands are complex systems that are not an appropriate solution for AMD remediation if not properly managed or understood (Johnson and Hallberg, 2002). A more fundamental understanding of the microbiological processes that are taking place within a wetland is therefore essential in order to better elucidate the mechanisms for improved remediation. This study aimed to enhance this understanding by quantifying microbial community dynamics within an AMD-impacted natural wetland, and to specifically address the hypothesis that differences in microbial community composition and metabolic activities of these communities can account for the remediation observed in the wetland.

**FIGURE 1:**
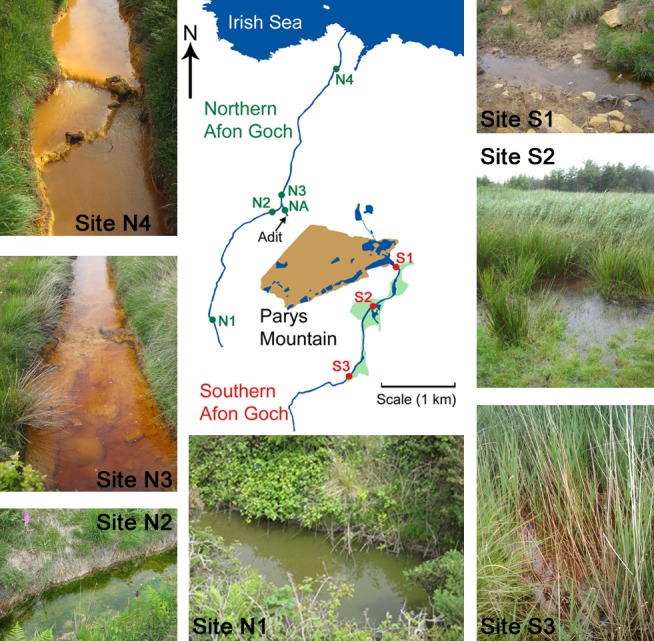
Sample sites within the Parys Mountain river catchment in Anglesey, Wales, United Kingdom. Sites on the southern Afon Goch river are marked S1–S3, with the wetland areas shaded in green. Sites on the northern Afon Goch river are marked N1–N4. Representative photographs of the samples sites along each river taken during times of sampling are shown. NA is the location of the adit that discharges AMD to the river.

The United Kingdom has a long history of metal mining leading to significant pollution problems as a result of AMD (Mayes et al., 2010). The Parys Mountain (Mynydd Parys) copper mine in Anglesey, North Wales, ceased operation in 1911 but accounts for some of the highest releases of Fe, Cu, and Zn nationally (Mayes et al., 2010). Significant volumes of AMD enter the small northern Afon Goch river via a mine adit (**Figure 1**). Following changes in drainage routes from the mine, this river has experienced substantially greater pollution in the last 15 years such that it is a recently impacted ecosystem that has not fully adapted to AMD with no abundance of wetland plants (Coupland and Johnson, 2004). AMD also enters a second river, the southern Afon Goch, which has experienced AMD pollution for over a century. While heavily polluted by dissolved metals and acidity at its source, there have been improvements to downstream water quality in this river due partly to remediation by a large natural wetland (Boult et al., 1994; Batty et al., 2006; Dean et al., 2013). This river system provides an ideal study site to quantify and contrast the microbial community composition in river sediment of a recently impacted ecosystem without wetland vegetation compared to a long-term impacted ecosystem with a substantial natural wetland, which is expected to have adapted to AMD pollution. In addition, by comparing the AMD affected wetland with a nearby unpolluted wetland site, we are able to examine changes in wetland microbial community structure on the basis of AMD exposure. This will allow us to test our hypothesis and examine whether there are differences in predicted metabolic activities derived from distinct bacterial communities found in wetland and river sediment sites.

## Materials and Methods

### Field Site Locations and Sampling

Water and sediment samples were collected from five sites along the northern Afon Goch river and three sites within the wetland through which the southern Afon Goch river flows (**Figure 1**). Samples were also taken at an unpolluted wetland (UW) site at Cefni Reservoir that is 13 km due South of Parys Mountain. Exact locations are provided in Supplementary Table S1. The northern Afon Goch rises to the west side of Parys Mountain and runs approximately 5.5 km in length with the Dyffryn Adda adit from the Parys Mountain mine joining at approximately 2.5 km along the river. The river then runs north through the town of Amlwch and enters the Irish Sea through an industrial site next to the port. The river course is largely canalized. Five sites were sampled (**Figure 1**) two upstream of the adit (site N1 and N2), the Dyffryn Adda adit itself (site NA), and two sites downstream of the adit (N3 and N4). Sampling was carried out on four (at site N1) or six (sites N2–N4 and NA) occasions on June 2010 (all sites), July 2010 (all sites), August 2013 (all sites), October 2013 (not site N1), March 2014 (all sites), and October 2014 (not site N1).

The southern Afon Goch is 11 km in length and runs south of Parys Mountain (**Figure 1**). Approximately 500 m below the mine source at the Mona adit, the river flows through a natural wetland of ∼0.1 km^2^. The river then runs east and enters the Irish Sea at Dulas Bay. Three sites were sampled within the wetland (sites S1–S3). Site S1 is an entry point for AMD runoff at the start of the wetland, while sites S2 and S3 are in the middle and at the end of the wetland, respectively (**Figure 1**). Sampling was carried out on four (site S2) or seven (sites S1 and S3) occasions on June 2010 (all sites), July 2010 (all sites), November 2011 (all sites), August 2013 (all sites), October 2013 (not site S2), March 2014 (all sites), and October 2014 (not site S2).

Triplicate sediment and water samples for analysis of pH, conductivity (as a measure of conductive ion concentrations), and dissolved, sediment, and particulate metals were collected at each site on each sampling occasion. A YSI 556 probe (Xylem Analytics, Letchworth, United Kingdom) was used to monitor water pH, conductivity, and dissolved oxygen. Samples for metal analysis (dissolved, sediment, and particulate) were collected at each site and processed within 5 h following collection. For dissolved metals, a known volume of river water (typically 100–200 mL) was filtered through a 0.45-μm cellulose acetate filter and the filtrate was stored in an acid-washed polypropylene bottle and was acidified to 2% with ultra-pure nitric acid. The pre-weighed filters were retained for analysis of metal particulates. Sediment samples were collected to approximately 1 cm depth using a plastic scoop and sealed in a plastic bag until analysis. On return to the laboratory, the filters containing suspended particulates were dried (60°C for 48 h), and the sediment samples were dried (60°C for 48 h) and then passed through a 250-μm filter. The particulate filter papers and 100 mg of sediment samples were digested in 67% ultra-pure nitric acid for 24 h at 100°C. Digests were then diluted to 2% nitric acid in deionized Milli-Q water (Millipore). Samples were refrigerated before metal concentrations in water samples and digests were analyzed by inductively coupled plasma atomic emission spectroscopy (ICP-AES) using a Perkin-Elmer Optima 5300 for Al, As, Cd, Cu, Fe, Mn, Pb, and Zn. The spectroscope was calibrated using an internal standard, which was a matrix matched serial dilution of Specpure multi element plasma standard solution 4 (Alfa Aesar).

For microbial community analysis, three sediment samples were collected from each site in March 2014 for DNA extraction. The southern Afon Goch wetland and Cefni Reservoir wetland are both dominated by *Juncus* sp. and samples were taken from sediment surrounding *Juncus* sp. roots from sites S1, S2, S3, and UW. In addition, a non-vegetated sediment sample was taken from site S1 (S1R), and a sediment sample surrounding cotton grass (*Eriophorum angustifolium*) roots was also taken from site S2 (S2C). Samples were taken in sterile 50-mL Falcon Tubes.

### DNA Extraction and 16S rRNA V3–V4 Gene Amplicon Sequencing

DNA was extracted from 100 mg sediment samples (three independent samples per site) using a Powersoil DNA isolation kit (MoBio Laboratories, Carlsbad, CA, United States) and quantified using a Nano-drop 3300 (Thermo-Scientific, Waltham, MA, United States). DNA from each site was pooled and then amplicons for Illumina MiSeq sequencing were generated from PCR reactions using primers Bakt_341F (S-D-Bact-0341-b-S-17) and Bakt_805R (S-D-Bact-0785-a-A-21) as previously described and validated (Herlemann et al., 2011; Klindworth et al., 2013), modified with Illumina overhang adaptors (forward overhang: 5’-TCG TCG GCA GCG TCA GAT GTG TAT AAG AGA CAG; reverse overhang: 5’-GTC TCG TGG GCT CGG AGA TGT GTA TAA GAG ACA G), to amplify the V3–V4 region of the 16S rRNA gene. PCR reactions were performed using KAPA HiFi HotStart Mix (KAPA, Woburn, MA, United States) and PCR amplification conditions of 95°C for 3 min, then 25 cycles of 95°C for 30 s, 55°C for 30 s, 72°C for 30 s, and then 72°C for 5 min. Following purification using AMPure XP beads (Beckman Coulter, High Wycombe, United Kingdom), index PCR and addition of Nextera sequence adapters was performed using a Nextera XT Index kit (Illumina Inc., San Diego, CA, United States) according to manufacturer’s instructions. Amplicons were sequenced using an Illumina MiSeq at the Genomic Technologies Facility, University of Manchester, and to a depth of over 100,000 sequences for each sample, and up to 1,473,986 for sample N4 (see **Table 1**). Sequence data were deposited in the European Nucleotide Archive (ENA), study accession number: PRJEB23187, sample accession numbers: ERS1983433 (site UW), ERS1983434 (site S1), ERS1983435 (site S2), ERS1983436 (site S3), ERS1983438 (site S2C), ERS1983439 (site N1), ERS1983440 (site N2), ERS1983441 (site NA), ERS1983442 (site N3), ERS1983443 (site N4), and ERS1983444 (site S1R).

**Table 1:** Summary of 16S rRNA gene amplicon sequence analysis and bacterial community diversity parameters for each sample site.

Site	Shannon–Weiner diversity index	Pielou’s evenness index	Chao1 species richness	Assigned taxa number^a^	Total OTUs^b^	Total sequences^c^
UW	7.35	0.58	22,592	1053	16,968	791,019
S1	5.73	0.45	9,865	721	5,863	367,239
S2	6.56	0.52	19,624	851	11,881	434,428
S3	7.92	0.63	24,969	1039	18,315	609,955
N1	8.01	0.63	25,675	1152	17,386	435,309
N2	7.15	0.57	16,862	1060	11,256	310,833
NA	4.29	0.34	5,283	463	3,678	542,593
N3	3.36	0.27	16,491	1102	13,582	1,106,448
N4	5.63	0.45	23,115	1009	21,438	1,473,986


*^*a*^Assigned taxa number indicates the number of distinct assigned taxa at genus level (excluding unassigned taxa) at each site.*

*^*b*^Total OTUs indicate the sum of all OTUs for each site and from all sequences.*

*^*c*^Total sequences indicate the number of sequence reads (sequence depth) after end-pairing and chimera removal for each site.*

### Statistical Analysis of Environmental Data

Principal component analysis (PCA) of environmental data was performed using the R vegan package v.2.4.2. The environmental data (except pH) was log-transformed prior to PCA. The PCA comprised a matrix of 46 datasets (replicates) from the six samples with 24 variables used. Statistical comparison of environmental data was performed using one-way ANOVA (*p* < 0.05) and Tukey’s multiple comparison *post hoc* test using GraphPad Prism. Hierarchical clustering (HC) of environmental data was performed in R and statistical comparison of nodes performed by a Similarity Profile (SIMPROF) test (Clarke et al., 2008).

### Sequence Data Analysis

Raw de-multiplexed sequence reads of the 16S rRNA V3–V4 region were trimmed and paired and further filtered using QIIME v.1.9.0 (Caporaso et al., 2010). Chimeric sequences were identified and removed by UCHIME v.4.2 (Edgar et al., 2011) before operational taxonomic units (OTUs) picking. OTUs were *de novo* picked at 97% similarity using UCLUST (Edgar, 2010). Taxonomic classification of the representative sequences was performed using the Greengenes v.13.8 97% OTU dataset and using the Naive Bayes machine learning classifier, which was trained using the amplified V3–V4 region sequence reads (Werner et al., 2011). In order to evaluate the microbial alpha diversity in each sample, Shannon–Weiner diversity index (*H*), Pielou’s evenness index (*H*/*H_max_*), and Chao1 species richness estimate values were calculated using QIIME. Before the calculations, samples were resampled to an even depth of 310,833 sequences per sample, the sequence depth size of the smallest sample (N2). Rarefaction curves were also generated by QIIME.

The assigned taxa dataset was statistically analyzed by HC and by non-metric multidimensional scaling (NMDS) both performed using R vegan package v.2.4.2. Taxa relative abundance was square-root-transformed and a distance matrix based on Bray–Curtis dissimilarity was obtained for HC. Complete linkage was used as an agglomerative clustering method and SIMPROF was used for identifying genuine groups between samples in HC. Samples were classified as “unpolluted” and “polluted” groups according to the environmental data. Analysis of similarity (ANOSIM; Clarke, 1993) was used to evaluate taxa assemblage difference between those groups and similarity percentage (SIMPER) analysis (Clarke, 1993) was used to identity taxa discriminating these groups and their contribution. BIO-ENV (Clarke and Ainsworth, 1993) was used to obtain the best subsets of environmental variables explaining taxon assemblage.

Metabolic structure was predicted from the 16S rRNA gene amplicon library of each sample site using the PAPRICA v.0.4.0 metabolic inference pipeline (Bowman and Ducklow, 2015). For this analysis, libraries were subsampled to 310,833 reads, the size of the smallest library. Abundance of enzymes and metabolic pathways were normalized according to the estimated number of 16S rRNA gene amplicon copies for each sample. For generating relative log_2_ fold-change abundance values, normalized enzyme abundance values were converted to logarithmic scale (base 2) after adding a value of 10 to allow consideration of zero values. HC of normalized metabolic pathway abundances was performed using the Euclidean distance method and heatmaps were created using R vegan package v.2.4.2.

## Results and Discussion

### Environmental Characterization

**FIGURE 2:**
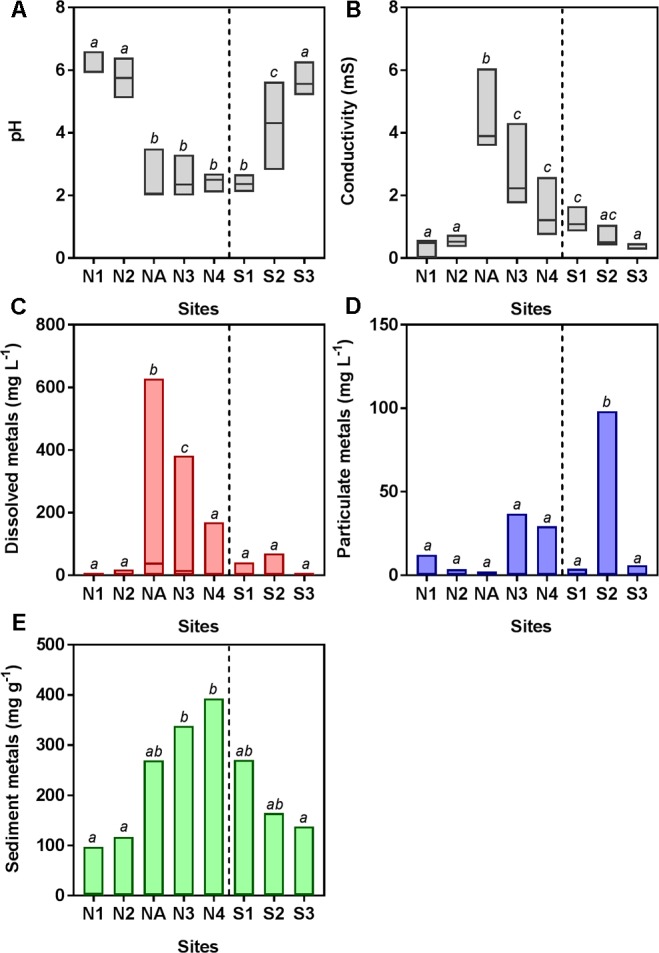
Water chemistry [**(A)** pH and **(B)** conductivity] and total metal concentration in water [**(C)** dissolved and **(D)** particulate] and **(E)** sediment samples. Data are pooled from triplicate analyses taken on four to seven sampling occasions (June 2010, July 2010, November 2011, August 2013, October 2013, March 2014, and October 2014). Boxes show the minimum and maximum values and the line within the boxes shows the median values. Boxes that do not share lowercase letters are significantly different (*p* < 0.05) as determined by one-way ANOVA. Data for individual metals (Al, As, Cd, Cu, Fe, Mn, Pb, and Zn) are shown in Supplementary Figure S1.

The southern and northern Afon Goch rivers are both exposed to AMD but have very different environmental characteristics, due in part to the presence of the natural wetland on the southern river, which is dominated by *Juncus* sp. and some *E. angustifolium* (cottongrass) and *Phragmites* sp. The northern Afon Goch has neutral pH and low conductivity water at sites N1 and N2 upstream of the adit entering the northern river, while highly acidic (pH 2.3–2.5), high conductivity water was downstream, although this was slightly diluted at site N4 (**Figure 2**). An increase of pH from 2.4 to 5.7 and a decrease of conductivity from 1.1 to 0.3 mS were observed along the wetland from site S1 to S3, indicative of the remediation process previously described (Dean et al., 2013). Dissolved metal concentrations within the water across the sites mirror the conductivity profile, and most dissolved metals show a similar profile across the sites with highest concentrations at site NA and N3 (**Figure 2C**). Many particulate metals including Al, Cu, Fe, Pb, and Zn show high concentrations within the middle of the wetland at site S2 (Supplementary Figure S1). This is likely due to the increase in pH causing increased precipitation of metals at site S2, in contrast to the higher concentration of dissolved metals, and lower particulate metals, within the acidic conditions at site S1. Overall, metal concentrations within the sediment are high in the acidic sites, particularly at N3 and N4 (**Figure 2E**) although sediment metal profiles vary depending on the element (Supplementary Figure S1). One unexpected pattern was the high concentration of some metals within sediment (particularly Mn, Pb, and Zn) at site N1 despite this otherwise unpolluted site being located upstream of the mine adit. This site can experience metal rich run-off from the mine site during high rainfall and is a slow-flowing, neutral pH section of the river, which will lead to greater sedimentation of metals.

**FIGURE 3:**
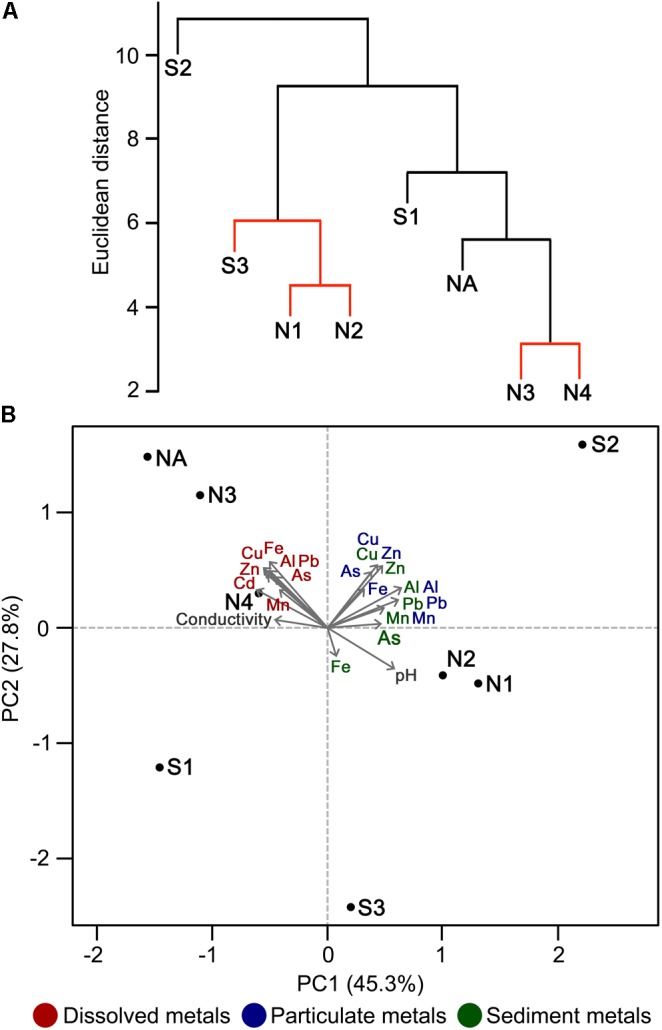
Discrimination of sites on the basis of physicochemical parameters. Hierarchical cluster analysis **(A)** and PCA ordination plot **(B)** illustrating the discrimination between sample sites according to environmental properties and their correlation with each environmental factor analyzed. In **(A)**, sites with non-significant clustering (*p* < 0.05) as determined by SIMPROF are indicated with red lines.

Principal component analysis indicates that the high concentrations of dissolved metals are key variables that correlate with the clustering of sites NA, N3, and N4, and to a lesser extent with site S1 (**Figure 3**). In contrast, the less polluted sites N1, N2, and S3 correlate with high water pH (pH 5.1–6.4), high concentration of Mn in sediments, and low concentration of As and Fe in sediments. S2 clustered separately from the other sites (**Figure 3A**) and correlates with particulate metals in the water column (**Figure 3B**). It was previously observed that the wetland remediation process is mediated in part by precipitation of metals in the middle of the wetland (Dean et al., 2013), as also seen here by the particulate metal profile (**Figure 2D**). Natural wetlands such as this one can function as long-term metal sinks by reducing flow rate, reducing acidity, and promoting microbial activities such as sulfate reduction (Beining and Otte, 1996; Webb et al., 1998; Dean et al., 2013). This activity gives rise to a subsequent immobilization of metals in the lower reaches of the wetland. In contrast, no significant change in particulate metals is observed downstream of the northern Afon Goch adit, which does not pass through any vegetation.

### Microbial Community Structure

**FIGURE 4:**
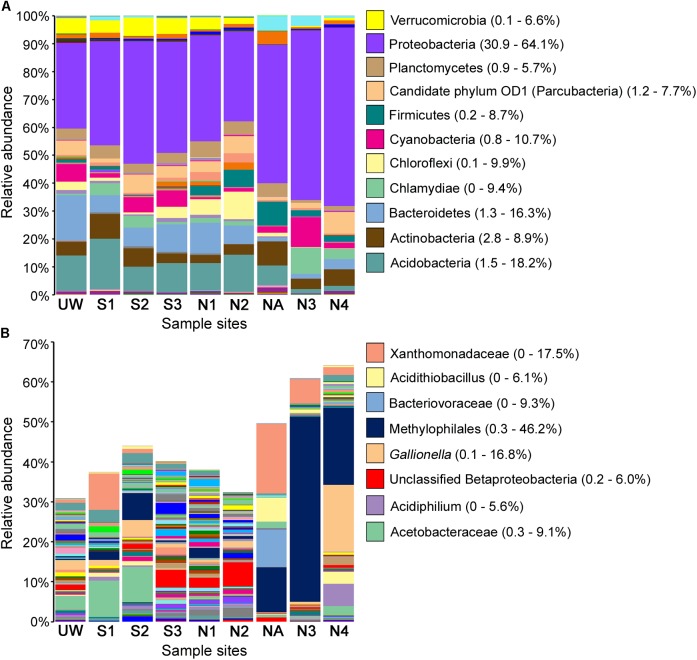
Relative abundance of bacterial taxa following OTU taxonomic assignment. All assigned taxa at phylum level where possible **(A)** and Proteobacteria shown to the highest resolution possible down to species level where possible **(B)** for each sample site. Selected taxa of high abundance in multiple samples are labeled, with ranges of relative taxa abundance given in parentheses.

DNA from sediment samples at all sites, as well as from an unpolluted wetland nearby (site UW; Supplementary Table S2) were used for 16S rRNA gene amplicon sequencing. Following processing 367,239–1,473,986 sequences were derived from each site, yielding 3678–21,438 total OTUs (**Table 1**). All samples showed a consistent level of saturation of OTU richness (Supplementary Figure S2). The adit (site NA) had the lowest OTU number whereas the equally highly polluted site N4 had the highest number of total OTUs. Likewise, both the Shannon–Weiner diversity index and Chao1 species richness estimate indicated that site NA had low bacterial diversity, while the Pielou’s index score indicated that this site had low community evenness. In contrast, unpolluted sites UW, N1, N2, and S3 were the most diverse. In particular, the Shannon–Weiner diversity score indicated that site S3 diversity was substantially improved compared to sites S1 and S2 at the lower reaches of the wetland. Yet the impacted wetland sites S1 and S2 maintained diversity (with scores of 5.73 and 6.56, respectively) compared to the AMD impacted river sites NA, N3, and N4 (with scores of 4.29, 3.36, and 5.63, respectively). This pattern was also seen with the Chao1 species richness and the evenness scores and demonstrates that the effect of pollution on species composition is greater in the river sediments than within the wetland. The reduced bacterial diversity at polluted river sites NA, N3, and N4 was also validated on the basis of OTU taxonomic assignments (Supplementary Data Sheet 1) and visualized in **Figure 4**. Excluding the unassigned group, the unpolluted sites UW, S3, N1, and N2 had between 1039 and 1060 unique assigned taxa at the genus level, while the number of taxa was substantially reduced at site NA (463; **Table 1**). At the start of the wetland (site S1), all diversity parameters were lower than for all other wetland sites (**Table 1**). Equivalent values were seen at an S1 site that was not directly associated with a wetland plant (site S1R; Supplementary Table S3). At site S2 in the middle of the wetland, a reduction in OTU number and diversity was observed in comparison to the unpolluted wetland sites S3 and UW. The presence of *Juncus* plants growing within the sediment (site S2) in contrast to cottongrass (site S2C) did not lead to substantial variation in bacterial diversity (Supplementary Table S3). Overall, the wetland maintains higher taxa richness despite a pollution gradient, unlike downstream of site NA where a decrease in bacterial diversity and an increase dominance of individual taxa was observed.

Proteobacteria, Actinobacteria, and Acidobacteria are normally highly abundant in most soils, with Proteobacteria typically representing 30–40% of all bacterial taxa (Roesch et al., 2007; Lauber et al., 2009). Likewise, in many different AMD environments, these bacterial taxa dominate despite overall low diversity (Chen et al., 2016). As expected, Proteobacteria was dominant in all of the sites, especially in NA, N3, and N4, where this phylum accounted for 49.6%, 60.9%, and 64.1% of the total taxon abundance, respectively (**Figure 4**). Examination of proteobacterial sequences in more detail showed dominance of an unclassified Xanthomonadaceae sequence particularly at site NA (17.5%) but also abundant at site S1, and an unclassified Methylophilales sequence with extremely high abundance (46.2%) at site N3 (**Figure 4B**). This unclassified Methylophilales taxon had reduced abundance at site N4 compared to N3; however, abundance of a *Gallionella* sp. was high (16.8%). Members of some of these taxa have previously been associated with Fe oxidation. For example, a Xanthomonadales taxon was observed in Fe(III)-rich sediments (Senko et al., 2008) and within acidophilic iron-oxidizing communities (Jones et al., 2015), although little is known about the organisms involved in this activity. *Gallionella* sp. is a bacterium with well-known iron oxidizing activity in microaerophilic environments (Mitsunobu et al., 2012) capable of generating biogenic iron oxides in sediments (Kikuchi et al., 2014). Although *Gallionella* spp. are commonly reported in pH neutral environments (Gault et al., 2012), they have been previously observed at acidic mine sites (Fabisch et al., 2013; Liljeqvist et al., 2015).

The relative abundance of Proteobacteria, including Xanthomonadales, Methylophilales, and Gallionellales, was much greater in northern Afon Goch sites NA, N3, and N4 compared to other sites, while Acidobacteria, Bacteroidetes, Planctomycetes, and Verrucomicrobia were less abundant in these sites (**Figure 4**). The dominant taxa observed within the wetland were equivalent to those seen previously in slightly acidic, non-metal polluted sediments of natural wetlands (Hartman et al., 2008; Peralta et al., 2013). However, taxa known to be involved in iron oxidation or sulfate reduction were not dominant within the near-surface sediment wetland samples analyzed here, suggesting that they are more abundant in other locations of the AMD ecosystem, such as deep anaerobic sediments, biofilms, or AMD mine water. For example, examination of a constructed wetland during its evolution over 225 days showed accumulation of sulfate reducing bacteria and aerobic bacteria in the water column over time (Diaby et al., 2015). Likewise, samples previously taken within the southern Afon Goch wetland at the sediment–water interface identified taxa that grouped phylogenetically with sulfate reducing and oxidizing bacteria (Dean et al., 2013).

### Factors Determining Microbial Community Structure

**FIGURE 5:**
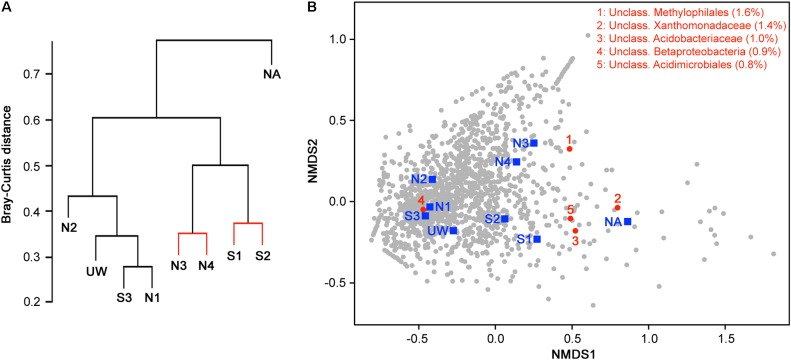
Discrimination of sites on the basis of bacterial community structure. **(A)** Hierarchical clustering of sample sites based on species similarity. Sites with non-significant clustering (*p* < 0.05) as determined by SIMPROF are indicated with red lines. **(B)** Two-dimensional NMDS plot of sites based on species similarity showing a separation between unpolluted sites (UW, S3, N1, and N2), polluted sites (S1, S2, N3, and N4), and the highly polluted mine adit site (NA) on the basis of NMDS1. Assigned taxa assemblage is shown (gray circles) and the top five taxa with the most contribution (% contribution) to site assemblage based on SIMPER analysis are indicated and listed in red.

Hierarchical clustering on the basis of community structure similarity found that unpolluted sites UW, N1, N2, and S3 clustered together, while a second cluster included sites S1, S2, N3, and N4, with the adit site NA as an outlier (**Figure 5A**). There was no significant distinction between S1 and S2 and between N3 and N4, but discrimination between wetland sites S1 and S2 compared to sites NA, N3, and N4 was significant (*p* < 0.05). On the basis of the physicochemical data, sites UW, S3, N1, and N2 were classified as an unpolluted group while sites S1, S2, N3, and N4 plus site NA were classified as a polluted group. ANOSIM demonstrated that there was a significant separation between the two groups (*R* = 0.61, *p* = 0.016) on the basis of community structure. NMDS ordination further demonstrated similar clustering of polluted and unpolluted sites (**Figure 5B**). Inclusion of the 1608 assigned taxa in the ordination indicated a greater density of taxa associated with the unpolluted sites, as shown by the taxa clustered on the left side of the plot. Of these taxa, 139 were uniquely present in the unpolluted group compared to 176 unique to the polluted group of sites. SIMPER analysis indicated that the unclassified Methylophilales taxon, which was dominant at sites NA, N3, and N4 (**Figure 4B**), was the highest ranked contributor to the assemblage of the sites (**Figure 5B**). A Methylophilales taxon associated with Cu tolerance was previously reported as one of the most abundant taxa within a microbial mat involved in natural remediation of heavy metal-contaminated mine water (Drewniak et al., 2016), while the same taxon was abundant in streams exposed to alkaline mine drainage (Bier et al., 2014). The other dominant taxa within the polluted sites were also shown to be key contributors to the pattern of site assemblage (**Figure 5B**).

Analysis of distinct AMD environments across the world has begun to identify dominant factors that influence species composition and diversity of microbial communities exposed to AMD (Méndez-García et al., 2015; Huang et al., 2016). For many cases, pH is a dominant factor for explaining variation in species diversity (Kuang et al., 2013; Liu et al., 2014). Indeed more generally, soil pH is often considered as a key determinant of bacterial community structure (Fierer and Jackson, 2006; Lauber et al., 2009). While we observed that pH was a key factor explaining the PCA clustering of the low pollution sites N1, N2, and S3 (**Figure 2B**), BIO-ENV analysis found that pH alone was not the key environmental variable for explaining the variation in community structure between the sites. Conductivity and metal concentration characteristics are strongly correlated with species assemblage, indicating that these factors may be particularly important (**Table 2**). Dissolved metal concentrations entering the northern Afon Goch river were extremely high (up to 628 mg L^-1^ of dissolved Fe at site NA) in comparison to many other studied AMD environments (Méndez-García et al., 2015), and this may in part explain the influence of conductivity and dissolved Fe and other metals as a key predictor of reduced species diversity here. Analysis of an acidic Teutonic Bore mine site in Australia also observed a strong correlation between conductivity and bacterial community structure, and this location also exhibited very high metal concentrations (Wakelin et al., 2012).

**Table 2:** Set of environmental variables from BIO-ENV analysis that best explain the assemblage of the bacterial community structure obtained from relative abundance of taxonomic assignments.

Variables	Rho
Conductivity, Dis(Fe), Dis(Al), Dis(Cu), Dis(Mn), Dis(As), Par(Fe), Sed(As)	0.854
pH, Dis(Fe), Dis(Al), Dis(Mn), Dis(As), Dis(Pb), Par(Fe), Sed(Fe), Sed(As)	0.854
pH, Conductivity, Dis(Fe), Dis(Zn), Dis(Cu), Dis(Mn), Dis(As), Dis(Pb), Par(Fe), Sed(Fe), Sed(As)	0.850
pH, Conductivity, Dis(Zn), Dis(Al), Dis(Mn), Dis(As), Dis(Pb), Par(Fe), Sed(Fe), Sed(As)	0.849
Conductivity, Dis(Zn), Dis(Cu), Dis(As), Dis(Pb), Par(Fe), Sed(As)	0.845
pH, Dis(Fe), Dis(Mn), Dis(As), Dis(Pb), Par(Fe)	0.841
pH, Dis(Cu), Dis(As), Dis(Pb), Par(Fe)	0.836
pH, Conductivity, Dis(Fe), Dis(Zn), Dis(Al), Dis(Cu), Dis(Mn), Dis(As), Dis(Pb), Par(Fe), Sed(Fe), Sed(As)	0.835
pH, Dis(As), Dis(Pb)	0.812
Dis(Fe), Dis(Al), Dis(As), Dis(Pb)	0.811


*Top 10 variables are shown. Dis, dissolved values; Par, particulate values; Sed, sediment values; Rho, Spearman’s rank correlation coefficient.*

While site clustering on the basis of taxa assemblage could be defined by the polluted and unpolluted groups, the wetland sites were nevertheless always significantly distinct from non-wetland river sites (**Figure 5A**). Organizing the sites as a river group and a wetland group allowed the comparison of assigned taxa on the basis of wetland plant presence. Most of the assigned taxa (73%) were shared between the river and wetland groups, while only 316 taxa were unique to the river group and 118 were unique to the wetland group. Some taxa had high abundance within wetland sites, but low abundance within non-wetland river sites (Supplementary Data Sheet 1b). These included an unclassified Bacteroidales (with an abundance of 9.8% at UW and 2.7% at S1 but 0.1% at N3 and N4) and an unclassified Acetobacteraceae (with an abundance of 8.9% and 8.7% at S1 and S2 but 0.3% at N1 and N2). Within the wetland sites, there was also a large dominance of taxa incorrectly classified by the Greengenes database as “Cyanobacteria,” but which include eukaryotic Stramenopiles that were identified via a chloroplast sequence, and so will slightly inflate the bacterial species richness values. This is probably due to the presence of diatom species, which we have previously identified from this wetland by 16S rRNA gene amplicon sequencing (Dean et al., 2013). Together, this suggests an increased dominance of photosynthetic microorganisms at the surface sediment of the wetland. In addition to plants, these oxygenic phototrophs will provide the system with oxygen and organic carbon to fuel heterotrophic activities, and will help drive biofilm production, which is an important location for sulfate metabolism to drive metal sulfide formation and metal precipitation (Roeselers et al., 2008; Chen et al., 2016).

### Prediction of Metabolic Potential

**FIGURE 6:**
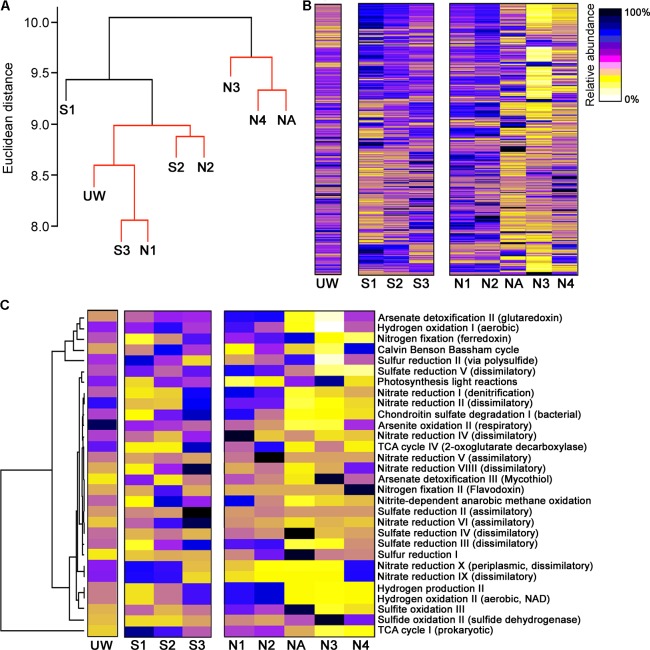
Metabolic prediction analysis and discrimination of sites on the basis of metabolic potential. **(A)** Hierarchical clustering of sample sites based on metabolic pathway similarity. Sites with non-significant clustering (*p* < 0.05) as determined by SIMPROF are indicated with red lines. Heat maps showing the predicted abundance of all prokaryotic metabolic pathways **(B)** and selected metabolic pathways related to element cycling **(C)**.

Although wetland plants directly promote some bulk extraction of dissolved metals into their tissues (Dean et al., 2013), the primary bioremediation action within the wetland will be due to microbial-derived enzymatic activities. For example, it is clear that there is substantial formation of particulate metals within the middle of the wetland (**Figure 2D**) likely due to altered biogeochemical processes at this location (Sobolewski, 1999; Dean et al., 2013), for example, the generation of insoluble compounds during oxidation processes (Hafeznezami et al., 2012) and sulfide compounds due to metal reduction (Wu et al., 2013), as well as redox and pH control to further influence metal precipitation (Frohne et al., 2011). We might therefore expect to see differences in functional enzymatic traits between the wetland sites and the AMD river sites. To quantify the microbial-derived metabolic potential (Kuang et al., 2016) of each site’s sediment, we used the PAPRICA metabolic inference prediction method by phylogenetic placement as an alternative to methodologies such as Tax4Fun and PICRUSt, which have some limitations for environmental microbiome analysis (Bowman and Ducklow, 2015; Koo et al., 2017). Clustering of each site on the basis of metabolic potential generated a profile that distinguished NA, N3, and N4 from the other sites (**Figure 6A**), due to a substantial predicted decrease in abundance of many enzymes specifically at these three sites within the ochre-rich sediment (**Figure 6B**). Metabolic and biochemical reactions with various elements including sulfur, arsenic, carbon, hydrogen, and nitrogen are important for adaptation by prokaryotes in AMD habitats (Méndez-García et al., 2015). The PAPRICA model inferred decreased abundance of many of these pathways within sites NA, N3, and N4, yet some reactions, such as sulfate and sulfur reduction, at site NA were predicted to have high abundance (**Figure 6C**). The overall metabolic prediction profile is particularly significant as this indicates a clear-cut distinction between the wetland and non-wetland AMD sites. The sediment at sites NA, N3, and N4 has very high Fe and As concentrations with high concentrations of other metals in the surface water. Therefore, we might speculate that the extreme metal-rich and acidic environment leads to inhibition of some enzymatic activities within the microbial communities. The reduced metabolic potential within these sites is not due to reduced bacterial abundance because the presented data are normalized on the basis of 16S rRNA gene amplicon sequence read number (approximately 300,000 reads taken from each site). In contrast, the metabolic potential remains stable within the wetland sediment sites despite AMD exposure. This clear difference in the metabolic potential of bacterial communities at the polluted wetland sites compared to the polluted river sites (**Figure 6A**) contrasts with the more subtle discrimination between these sites on the basis of bacterial community structure (**Figure 5A**). We therefore suggest that wetland environments with their associated AMD impacts influence the metabolic structure of sediment microbial communities. These selected communities are sufficiently tolerant of these conditions that the microbial mediated processes of immobilization of metals in the sediment are sustained during the wetland evolution.

**FIGURE 7:**
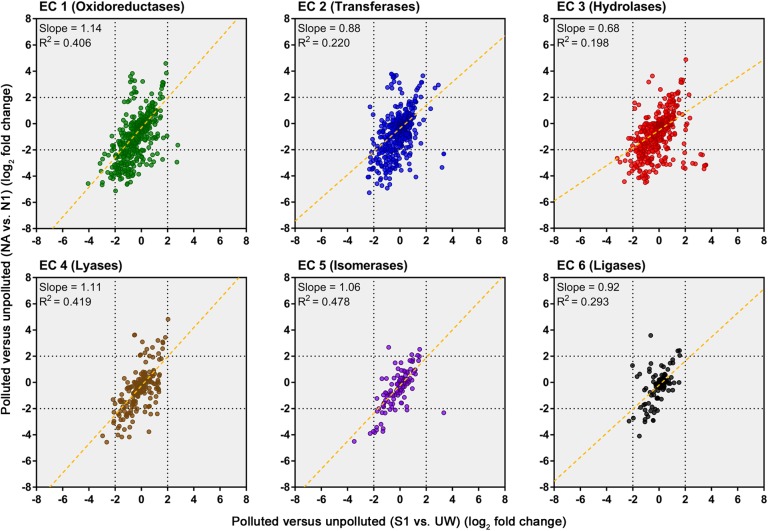
Predicted changes in enzyme abundance in response to AMD pollution. Scatter plots of Enzyme Commission (EC) enzyme reaction classes showing the relationships between wetland (southern Afon Goch) and non-wetland (northern Afon Goch) log_2_ fold-change values for polluted (site S1 or NA) versus unpolluted (site UW or N1) sites. Each dot corresponds to log_2_ fold-change in abundance of a predicted enzyme reaction. The linear regression fit line is plotted.

**FIGURE 8:**
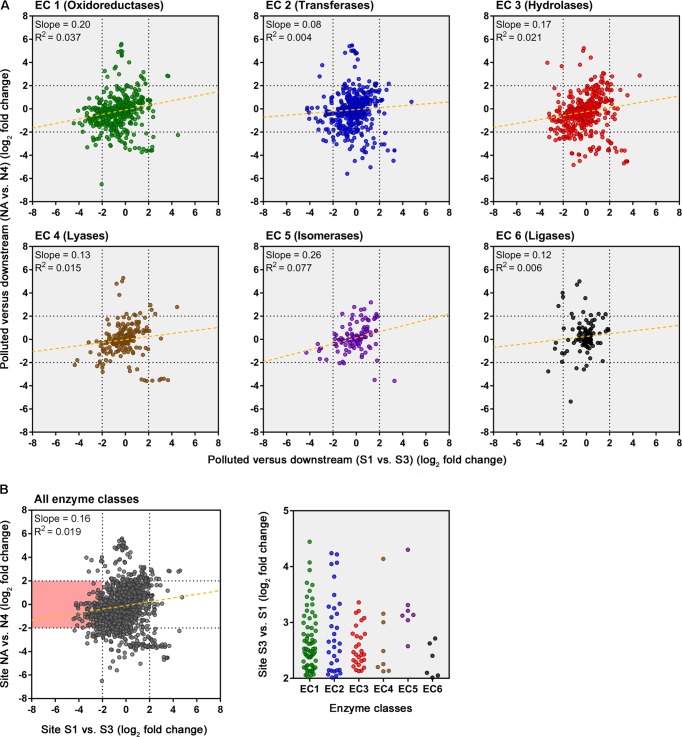
Predicted changes in enzyme abundance in response to the presence of a wetland. **(A)** Scatter plots of EC enzyme reaction classes showing the relationships between wetland (southern Afon Goch) and non-wetland (northern Afon Goch) log_2_ fold-change values for pollution source (site S1 or NA) versus downstream sites (site S3 or N4). Each dot corresponds to log_2_ fold-change in abundance of a predicted enzyme reaction. The linear regression fit line is plotted. **(B)** Scatter plot for all enzyme classes. The red-shaded quadrant indicates enzymes that are significantly increased in abundance specifically at site S3 but not at site N4, relative to the pollution source sites. The enzymes of each class that are increased in abundance at site S3 are shown (right plot).

An evaluation of the predicted change in abundance of enzyme classes between polluted and unpolluted sites on the southern Afon Goch (site S1 relative to site UW) and northern Afon Goch (site NA relative to site N1) was performed (**Figure 7**). For most enzyme classes, there was a relatively strong positive correlation (*R*^2^ > 0.4) in predicted enzyme abundance change between the northern and southern river sites, such that the majority of the enzymes that increased or decreased in abundance at site NA also increased or decreased at site S1. A second analysis compared changes along each river system, between the site of highest source of AMD pollution (site S1 or NA) and the furthest downstream site (site S3 or N4). This provides an evaluation of metabolic potential change moving through the wetland (*x*-axis) or moving down the northern Afon Goch river (*y*-axis; **Figure 8A**). These plots displayed no significant correlation (all *R*^2^ < 0.1) indicating that there are very different profiles of metabolic potential depending on whether the flow is through a wetland or not. Using >2 and <-2 log_2_ fold-change as the threshold of significant enzyme abundance change, the plots predict that there are a number of significant increases and decreases in enzyme abundance through the wetland with no change along the northern river, and likewise predicted changes in enzyme abundance along the northern river that are not seen through the wetland (**Figure 8A**). A number of significant increases or decreases in enzyme abundance (particularly with EC1, EC2, EC3, EC4, and EC6 enzymes) are specific to the northern river and result in no significant change through the wetland. In contrast, a few enzymes, especially of the EC1 oxidoreductase and EC2 transferase classes, have increased abundance through the wetland but are not predicted to increase abundance between site NA and N4 (**Figure 8B**). This prediction is in line with expected activities within AMD impacted wetland sediment, such as mechanisms that direct the oxidation of Fe(II) (Sobolewski, 1999; Johnson and Hallberg, 2005; Sheoran and Sheoran, 2006), although such iron oxidation mechanisms are poorly understood and therefore challenging to predict (Ilbert and Bonnefoy, 2013). A cluster of EC3 hydrolytic enzymes are also predicted to increase abundance through the wetland (**Figure 8B**). Breakdown and assimilation of organic carbon products, such as cellulose and other polysaccharides from plants, will likely require increased hydrolytic activities (Shackle et al., 2000), while metal hydrolysis reactions will take place alongside oxidation reactions to remove metals from solution (Sobolewski, 1999). As metal toxicity is reduced through the wetland, metal and acidic tolerance and detoxification mechanisms are less required for survival of the microbial communities. We suggest that the observed metabolic profile indicates differential exposure of the microbiota to metal stress. However, it should be noted that these methods of metabolic prediction, such as PAPRICA, Tax4Fun, and PICRUSt, may miss some functional genes, including metal tolerance genes, which are carried on plasmids.

## Overview and Conclusion

Wetlands provide significant ecological functions and can provide beneficial ecosystem services such as promoting bioremediation of contaminated water, which may be replicated in constructed wetlands (Zedler and Kercher, 2005; Sheoran and Sheoran, 2006; Vymazal, 2011). The potential for AMD bioremediation by wetlands derives from biogeochemical processes, which are enhanced by microbial activities (Jacob and Otte, 2003; Hafeznezami et al., 2012; Wu et al., 2013). While some of the processes controlling microbial community structure are beginning to be understood in natural wetlands with differing land use histories, and in constructed wetlands in response to wastewater inputs (Hartman et al., 2008; Peralta et al., 2013; Ansola et al., 2014), this is the first study of which we are aware to explore microbial community structure dynamics within a natural wetland exposed to AMD.

We show that a long-term AMD-impacted river, which has established a pollution-adapted wetland ecosystem, is clearly distinguished from a less adapted, non-vegetated river on the basis of variation in microbial community structure and hence predicted function. AMD river sediment in the absence of a wetland is characterized by the prediction of significantly reduced metabolic activity, indicative of stress response, while more specific metabolic changes are predicted within the wetland and which contribute toward the reduction in acidity and removal of dissolved metals. Our work suggests that wetlands can maintain high sediment microbial community diversity despite high levels of acidity and metal pollution, and this microbial community structure will in turn influence biogeochemical activities that can take place within the wetland. Future investigations of these biogeochemical processes will validate the predictions of the metabolic modeling by functional analysis. Furthermore, examination of other AMD impacted natural wetlands and constructed wetlands will be needed to identify conserved functional traits within these systems, and in particular determine whether characteristics of a long-established wetland ecosystem are also seen or can be replicated in a recently developed constructed treatment wetland.

## Data Availability

The datasets generated for this study can be found in the European Nucleotide Archive (ENA), Study accession number: PRJEB23187 (https://www.ebi.ac.uk/ena).

## Author Contributions

OA, AM, AD, and JP generated the data and performed the data analysis. KW, AD, and JP conceived the project. OA, KW, AD, and JP interpreted the results and wrote the paper. All authors read and approved this manuscript.

## Conflict of Interest Statement

The authors declare that the research was conducted in the absence of any commercial or financial relationships that could be construed as a potential conflict of interest.
