# Supplementary material for: Microbial Community Shifts in Response to Acid Mine Drainage Pollution Within a Natural Wetland Ecosystem
Source: Front Microbiol. 2018 Jun 27;9:1445. doi: 10.3389/fmicb.2018.01445 (PMC6036317; doi:10.3389/fmicb.2018.01445)
Supplement: Supplementary file 1 [file Table_1.PDF]

SUPPLEMENTARY TABLE S1. Locations of sampling sites on the southern Afon Goch (S1 – S3) and northern Afon Goch (N1 – N4, and NA) in Anglesey, North Wales, UK, and at an unpolluted wetland site (UW) elsewhere in Anglesey.

Sample site	Description	Location
S1	Start of wetland immediately below source of river	Lat. 53°23'13.5" N, Long. 4°19'31.9" W
S2	Within natural wetland	Lat. 53°22'54.7" N, Long. 4°19'42.4" W
S3	At end of wetland	Lat. 53°22'17.1" N, Long. 4°20'12.4" W
N1	Upstream of Dyffryn Adda adit	Lat. 53°22'52.8" N, Long. 4°22'02.0" W
N2	Upstream of Dyffryn Adda adit	Lat. 53°23'37.5" N, Long. 4°21'22.4" W
NA	Dyffryn Adda adit	Lat. 53°23'42.3" N, Long. 4°21'02.9" W
N3	Downstream of Dyffryn Adda adit	Lat. 53°23'47.2" N, Long. 4°21'03.6" W
N4	Downstream of Dyffryn Adda adit	Lat. 53°24'47.6" N, Long. 4°20'21.7" W
UW	Cefni Reservoir, non-AMD wetland site	Lat. 53°15'57.8" N, Long. 4°21'0.6" W
