# Supplementary material for: Microbial Community Shifts in Response to Acid Mine Drainage Pollution Within a Natural Wetland Ecosystem
Source: Front Microbiol. 2018 Jun 27;9:1445. doi: 10.3389/fmicb.2018.01445 (PMC6036317; doi:10.3389/fmicb.2018.01445)
Supplement: Supplementary file 2 [file Table_2.PDF]

SUPPLEMENTARY TABLE S2. Selected physicochemical parameters of water (pH, conductivity and dissolved metals) and sediment samples (metals) at the unpolluted wetland site (site UW) at Cefni Reservoir, Anglesey, UK. Data are mean values (± SEM) from 3 samples. BDL, below detectable limit.

**Parameter**	**Value**
pH	7.5 ± 0.1
Conductivity	0.49 ± 0.05 mS
Dissolved Al	0.12 ± 0.02 mg L^{-1}
Dissolved As	BDL
Dissolved Cd	BDL
Dissolved Cu	0.08 ± 0.04 mg L^{-1}
Dissolved Fe	0.29 ± 0.04 mg L^{-1}
Dissolved Mn	0.43 ± 0.19 mg L^{-1}
Dissolved Pb	BDL
Dissolved Zn	0.14 ± 0.03 mg L^{-1}
Sediment Al	16.06 ± 0.92 mg g^{-1}
Sediment As	0.01 ± 0.002 mg g^{-1}
Sediment Cd	0.90 ± 0.06 µg g^{-1}
Sediment Cu	0.01 ± 0.001 mg g^{-1}
Sediment Fe	32.54 ± 1.85 mg g^{-1}
Sediment Mn	4.11 ± 0.45 mg g^{-1}
Sediment Pb	0.02 ± 0.001 mg g^{-1}
Sediment Zn	0.15 ± 0.004 mg g^{-1}
