# Supplementary material for: Microbial Community Shifts in Response to Acid Mine Drainage Pollution Within a Natural Wetland Ecosystem
Source: Front Microbiol. 2018 Jun 27;9:1445. doi: 10.3389/fmicb.2018.01445 (PMC6036317; doi:10.3389/fmicb.2018.01445)
Supplement: Supplementary file 3 [file Table_3.PDF]

SUPPLEMENTARY TABLE S3. Bacterial community diversity parameters and 16S rRNA sequence analysis summary for additional samples at sites S1 and S2. S1, sediment associated with *Juncus* sp.; S1R, sediment from river without plant; S2, sediment associated with *Juncus* sp.; S2C, sediment associated with cottongrass sp.

Site	Shannon-Weiner diversity index	Pielou's evenness index	Chao1 species richness	Assigned taxa number[a]	Total OTUs[b]	Total sequences[c]
**S1**	5.73	0.45	9,865	721	5,863	367,239
**S1R**	5.71	0.45	10,395	731	6,670	667,578
**S2**	6.56	0.52	19,624	851	11,881	434,428
**S2C**	7.22	0.57	17,743	887	12,217	609,736

[a]Assigned taxa number indicates the number of distinct assigned taxa at genus level (excluding unassigned taxa) at each site.
[b]Total OTUs indicate the sum of all OTUs for each site and from all sequences.
[c]Total sequences indicate the number of sequence reads (sequence depth) after end-pairing and chimera removal for each site.
