# Supplementary material for: Microbial Community Shifts in Response to Acid Mine Drainage Pollution Within a Natural Wetland Ecosystem
Source: Front Microbiol. 2018 Jun 27;9:1445. doi: 10.3389/fmicb.2018.01445 (PMC6036317; doi:10.3389/fmicb.2018.01445)
Supplement: Supplementary file 5 [file Image_1.PDF]

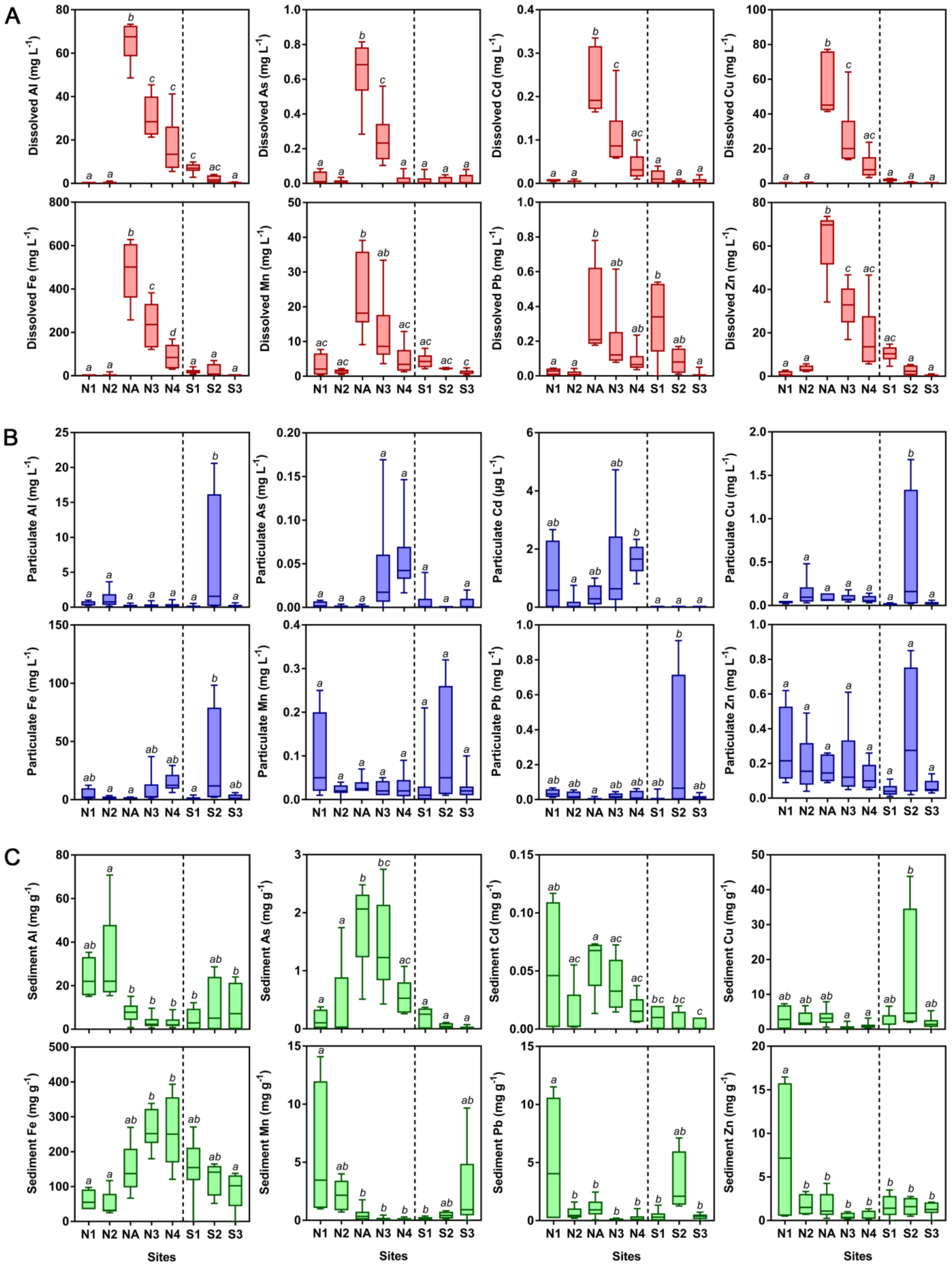

SUPPLEMENTARY FIGURE S1. Water and sediment metal concentrations taken at sites within the northern and southern Afon Goch (see Figure 1 for locations). Values of dissolved metals **(A)**, particulate metals **(B)** and sediment metals **(C)**. Data are pooled from triplicate analyses taken on 4 – 7 sampling occasions (between June 2010 and October 2014). Boxes show the 25th and 75th percentiles, the line within the boxes shows the median values. Whisker bars show the minimum and maximum values. Boxes that do not share lowercase letters are significantly different ($p < 0.05$) as determined by one-way ANOVA.
