# Supplementary figures and images for: Microbial Community Shifts in Response to Acid Mine Drainage Pollution Within a Natural Wetland Ecosystem
Source: Front Microbiol. 2018 Jun 27;9:1445. doi: 10.3389/fmicb.2018.01445 (PMC6036317; doi:10.3389/fmicb.2018.01445)

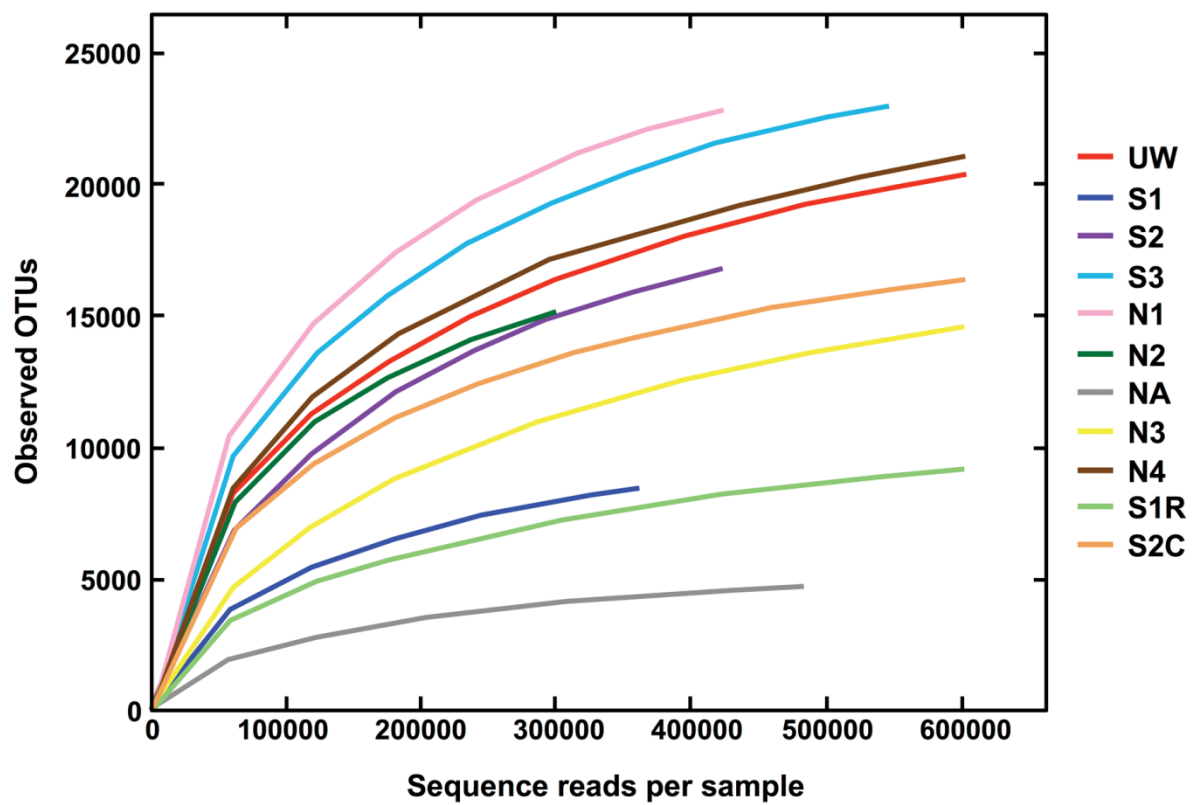

**SUPPLEMENTARY FIGURE S2.** Rarefaction curves of number of observed OTUs for each sample.

Supplement: Supplementary file 6 [file Image_2.PDF]
